# Magic and cognition: a scoping review of empirical evidence on cognitive and neural processes

**DOI:** 10.3389/fpsyg.2026.1921905

**Published:** 2026-09-03

**Authors:** Miguel De Lucas, Jon Andoni Duñabeitia

**Affiliations:** Centro de Investigación Nebrija en Cognición (CINC), Universidad Nebrija, Madrid, Spain

**Keywords:** covert attention, illusionism, magic, memory, misdirection, scoping review, surprise, visual perception

## Abstract

**Introduction:**

Magic, understood as a set of effects designed to produce experiences of impossibility, offers a distinctive methodological potential for cognitive science because it makes it possible to manipulate, with ecological validity, processes such as attention, perception, reasoning, and memory. Despite increasing research interest over the last two decades, the available evidence has not been systematically mapped across cognitive domains, methodological approaches, and the neural correlates underlying magic perception or processing. The aim of this scoping review was to map and synthesize the empirical evidence on the relationship between illusionism and human cognitive processes across four cognitive domains: attention and misdirection, visual perception, high-level cognition, and epistemic emotion and memory. Neural correlates of the magical experience were examined as a fifth synthesis category.

**Methods:**

A systematic search was conducted in Web of Science, Scopus, PubMed, and APA PsycINFO, with no date restriction, and reported following PRISMA-ScR guidelines. Of the 1,633 records identified, 55 studies met the eligibility criteria. Findings reported in these studies were mapped and synthesized narratively.

**Results:**

The most consistent findings concerned the attentional domain, indicating that covert attention contributes substantially to misdirection across several experimental contexts, with convergent findings reported by multiple laboratories. With regard to high-level cognition, the reviewed evidence does not clearly support the idea that magicians possess privileged psychological knowledge. In epistemic emotion and memory, responses to physically impossible events are related to the early development of intuitive physical knowledge. At the neural level, the limited evidence available suggests that observing magical effects involves networks associated with conflict detection, predictive processing, and expectation violation, including frontal regions (DLPFC, ACC, IFG) and the caudate nucleus, providing a preliminary biological correlate to the cognitive phenomena described above.

**Discussion:**

Overall, the findings support the view that magic constitutes a valid and promising methodological tool for the study of human cognition, although the heterogeneity of the studies explored so far and the geographical concentration of the research limit the generalizability of the conclusions.

## 1 Introduction

Magic is one of the oldest cultural practices based on the systematic creation of impossible experiences. In this article, the terms magic and illusionism are used interchangeably to refer to the set of effects and techniques designed to produce in the observer the experience of an impossible or inexplicable event. Although traditionally understood as a performing art, magic has increasingly attracted the attention of cognitive scientists because it offers a unique way to study how the human mind constructs, updates, and sometimes misinterprets reality (e.g., Rensink and Kuhn, 2015). A magic trick is not merely an entertainment device; it is a carefully designed cognitive event in which attention, perception, expectation, memory, causal reasoning, and emotional appraisal are manipulated to produce the experience of impossibility (Kuhn et al., 2009; Parris et al., 2009; Danek et al., 2014).

From a cognitive perspective, magic is especially valuable because it operates at the intersection between bottom-up sensory information and top-down expectations, a principle that has been formalized through Bayesian predictive-coding frameworks applied specifically to magic tricks (Grassi and Bartels, 2021). Magical effects often depend on diverting attention away from relevant actions, exploiting perceptual limitations, violating causal assumptions, or creating a gap between what spectators see and what they infer. This makes magic a promising experimental paradigm for investigating attentional control, misdirection, inattentional blindness, change blindness, perceptual prediction, causal reasoning, insight, curiosity, surprise, or disbelief, among others (e.g., Barnhart and Goldinger, 2014; Cui et al., 2011; Kuhn and Rensink, 2016; Macknik et al., 2008; Parris et al., 2009).

This capacity to manipulate cognitive processes in a controlled way has fostered a growing empirical research program with multiple lines of investigation that use magic tricks and magical stimuli to examine human cognition (Rensink and Kuhn, 2015). One of these lines of studies has mainly focused on attention and misdirection, analyzing how gaze cues, hand movements, social signals, and contextual expectations determine what observers notice or fail to notice (e.g., Kuhn et al., 2009; Cui et al., 2011; Barnhart and Goldinger, 2014). Other studies have examined visual perception, including perceptual prediction, representational momentum, and the conscious experience of disappearance or illusory motion (Kuhn and Rensink, 2016). Beyond perception, another research line has used magic in problem-solving research, in which participants attempt to infer the hidden method responsible for an apparently impossible effect, thereby allowing researchers to examine insight, fixation, explanation generation, metacognition, and causal reasoning (e.g., Danek et al., 2014; Olson et al., 2015a,b). More recent work has investigated epistemic emotions (namely, the affective responses associated with the pursuit, acquisition, or revision of knowledge, such as curiosity, interest, and surprise) using validated collections of magic-trick videos (Ozono et al., 2021; Marascia et al., 2026). In parallel, the available neuroimaging and electrophysiological studies have begun to identify the neural correlates involved in observing magic tricks and in processing violations of expectation (e.g., Danek et al., 2015; Lee et al., 2022; Parris et al., 2009).

Despite this growing interest, the empirical literature remains fragmented. Studies vary widely in their designs, populations, stimuli, outcome measures, and theoretical frameworks. This heterogeneity makes it difficult to obtain an integrated view of what has been learned from magic-based research and how this evidence contributes to cognitive science. Therefore, the aim of this scoping review was to map and synthesize the available empirical evidence on the relationship between magic and human cognitive processes. Specifically, the review addressed two complementary overarching questions: (1) which cognitive processes have been empirically investigated using magical stimuli? and (2) which neural correlates and neurocognitive outcomes have been reported in studies involving the observation, experience, or practice of magic? To address these questions, the included studies were organized into four categories corresponding to four cognitive domains, and one additional synthesis category regarding the neural correlates: attention and misdirection (D1), visual perception (D2), high-level cognition (D3), epistemic emotion and memory (D4), and neural correlates (D5).

The systematic search identified 55 original empirical studies published between 1995 and 2026, with a notable concentration of publications from 2010 onward. The studies were distributed across the four cognitive domains (D1, *n* = 17; D2, *n* = 8; D3, *n* = 15; D4, *n* = 10), and one additional neural category (D5, *n* = 5). The following sections present the findings organized first by cognitive domain and then by neural correlates.

## 2 Methods

### 2.1 Design, guidelines, information sources and search strategy

This scoping review was reported in accordance with the PRISMA extension for Scoping Reviews (PRISMA-ScR; Tricco et al., 2018), using a systematic search and selection process to map the available empirical evidence. Given the methodological heterogeneity of the included studies, a narrative synthesis was planned after data charting and thematic narrative mapping following the JBI manual for Scoping Reviews. The initial literature search was conducted in three electronic databases: Web of Science, Scopus, and PubMed on 28 May 2026. A total of 1,535 records were identified: 442 from Web of Science, 483 from Scopus, and 610 from PubMed. These records were imported into Rayyan.ai for duplicate detection, title and abstract screening, and full-text assessment (Ouzzani et al., 2016). After duplicate removal, 994 records remained for title and abstract screening. An additional retrospective verification search, using a database-adapted version of the original search strategy, was conducted in APA PsycINFO on 15 July 2026 and yielded 98 records. These records were cross-checked against the original search results by comparing titles, authors, and publication years. All 98 PsycINFO records were identified as duplicates of records already captured in the original three-database search. Therefore, the total number of records identified across the four databases was 1,633. After removing 639 duplicates, 994 records remained for title and abstract screening. The complete search strings for each database are provided in Supplementary Table S1.

### 2.2 Eligibility criteria and study selection process

**Figure 1:**
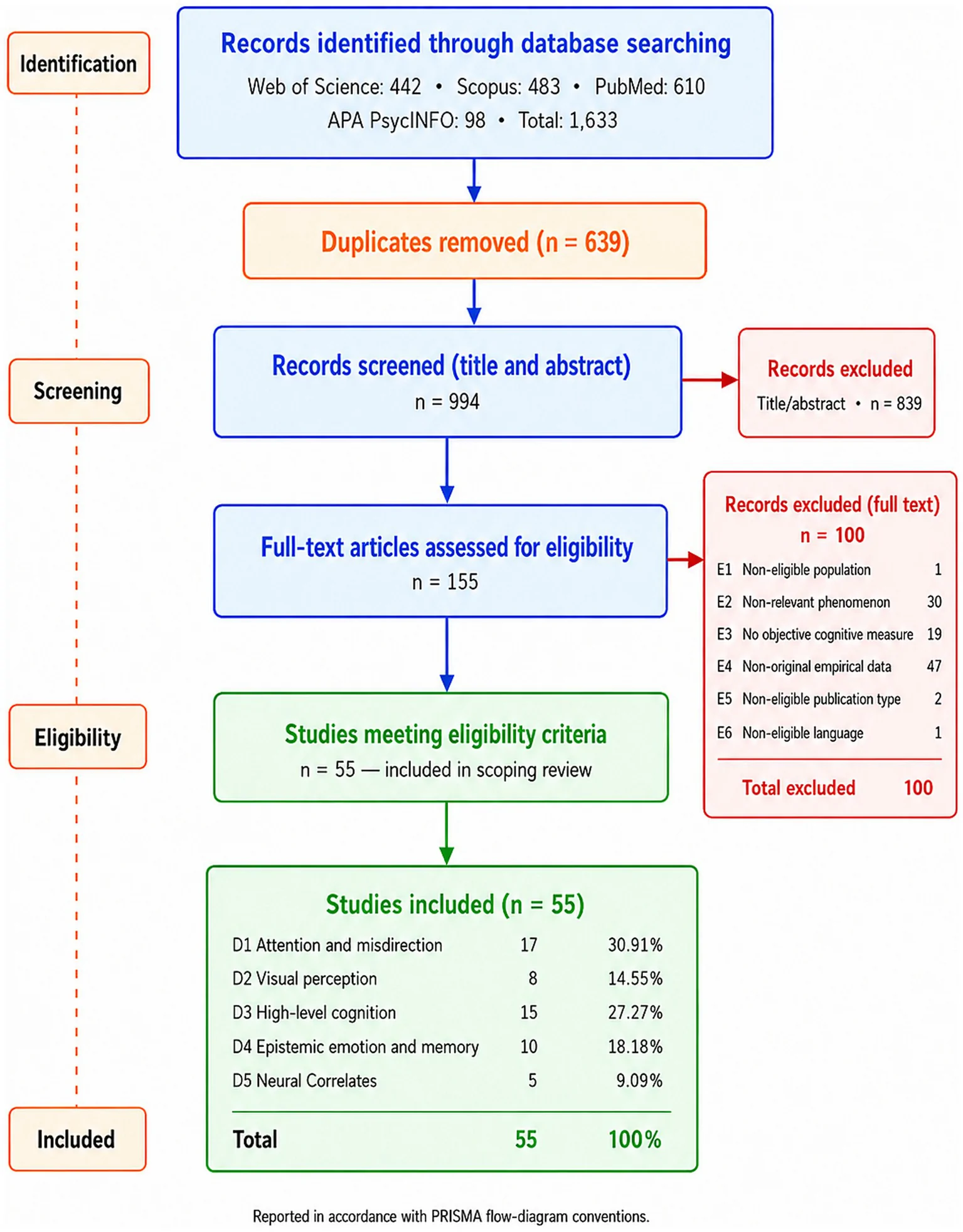
PRISMA-ScR flow diagram of the study selection process.

Eligibility criteria were defined using the PCC framework, considering participants, concept and context. Original empirical studies involving human participants were included when participants were exposed to magic tricks, magical stimuli, magic-based tasks, or magic-based research paradigms, and when the study reported outcomes related to cognitive, neurocognitive, perceptual, attentional, emotional, mnemonic, metacognitive, or neural processes. Eligible designs included experimental, quasi-experimental, observational, cross-sectional, normative, psychophysical, behavioral, neuroimaging, electrophysiological, and mixed empirical studies. No restrictions were established regarding age, population type, presence of a control group, or year of publication. No language filters were applied during the database searches. At full-text assessment, however, one article was excluded because its full text could not be reliably evaluated in a language accessible to the review team; this pragmatic restriction was coded as E6 (see Figure 1).

Studies without original empirical data, theoretical articles, reviews, meta-analyses, purely instructional works on magic, studies in which magic appeared only as an anecdotal or artistic element, and articles whose primary outcome was not related to cognition or neurocognition were excluded. Conference proceedings and conference-related documentation were also excluded. The complete inclusion and exclusion criteria applied during title and abstract screening and full-text assessment are presented in Table 1.

**Table 1:** Inclusion and exclusion criteria according to the PCC framework.

PCC component	Inclusion criteria	Exclusion criteria
Participants	Empirical studies with human participants of any age exposed to magic tricks, magical stimuli, magic-based tasks, or experimental paradigms related to magic. General, child, adult, educational, clinical, or specific populations were included whenever the study assessed cognitive, neurocognitive, perceptual, emotional, mnemonic, or neural processes.	Studies without human participants; studies focused exclusively on the professional training of magicians without cognitive or neurocognitive variables; or works in which the population was not relevant to the review questions.
Concept	Studies using magic tricks, magical stimuli, illusionism, magic performances, magic videos, live magical effects, or magic-based experimental paradigms. Studies in which participants observed, interpreted, solved, evaluated, learned, or interacted with magical effects were included. Studies reporting empirical data related to cognition, neurocognition, attention, misdirection, perception, visual awareness, change blindness, inattentional blindness, memory, curiosity, surprise, interest, insight, causal reasoning, explanation generation, metacognition, decision-making, expectation violation, disbelief, or neural correlates.	Studies in which magic appeared only as an anecdotal, historical, artistic, commercial, or pedagogical element without empirical assessment of cognitive processes. Studies using non-magical illusions were excluded unless the stimuli were explicitly framed as magic or as a magic-performance-based paradigm. Studies whose primary outcome was unrelated to cognition or neurocognition. Works focused only on entertainment, motor performance, artificial intelligence, computational design, educational satisfaction, clinical symptoms, or artistic appreciation were excluded unless they contained relevant empirical data on cognitive, epistemic-emotional, perceptual, or neural processes.
Context	Original empirical studies, including experimental, quasi-experimental, observational, cross-sectional, normative, psychophysical, behavioral, neuroimaging, electrophysiological, and mixed designs.	Reviews, systematic reviews, meta-analyses, theoretical articles, opinion articles, editorials, non-empirical chapters, purely instructional works on magic, non-peer-reviewed literature, and studies without sufficient empirical relevance to the review questions.

After duplicate removal, 994 records were screened by title and abstract, and 839 of them were excluded. A total of 155 articles were assessed in full text. At this stage, 100 articles were excluded for the following explanatory reasons: E1, non-eligible population (*n* = 1); E2, non-relevant phenomenon (*n* = 30); E3, no eligible cognitive, neurocognitive, perceptual, emotional, mnemonic, metacognitive, or neural outcome (*n* = 19); E4, study without original empirical data, such as theoretical reviews, essays, or opinion articles (*n* = 47); E5, non-eligible publication type, such as conference proceedings, non-scientific articles, or other formats not suitable for peer review (*n* = 2); and E6, full text not assessable in a language accessible to the reviewers (*n* = 1). Finally, 55 studies met the eligibility criteria and were included in the scoping review.

### 2.3 Data extraction and categorization

Data were extracted using a structured codebook designed specifically for this review, covering 35 fields per study (32 content fields, including bibliographic information, study design, sample characteristics, type of magical stimulus, cognitive variables assessed, measurement instruments, main results, effect sizes when reported, methodological limitations, and funding sources; and 3 administrative fields recording extraction date, extractor identity, and methodological notes). The complete codebook is provided in Supplementary Table S2.

Narrative extraction forms were also prepared for all included studies in order to support the qualitative synthesis and ensure traceability between the codebook and the interpretation of findings. Each included study was assigned to a single primary synthesis category: D1, attention and misdirection; D2, visual perception; D3, high-level cognition; D4, epistemic emotion and memory; and D5, neural correlates. Assignment to domains was based on each study’s primary research question and principal outcome measures, rather than on the type of magic trick used or on secondary variables. When a study addressed more than one cognitive process, it was assigned to the domain corresponding to its main analytic focus. D5 was treated separately because it was defined by the level of measurement (i.e., neural or neurocognitive evidence) rather than by a single cognitive process, and therefore potentially intersected with each of the four functional domains. Given the number of studies within each domain, the narrative synthesis in the Results section highlights representative studies that illustrate the main empirical patterns identified. The complete list of the 55 included studies, together with their assigned synthesis category and full bibliographic reference, is provided in Supplementary Table S4.

### 2.4 Assessment of methodological quality and synthesis strategy

A critical appraisal of the study design of the included articles was done preliminarily using tools and criteria adapted to each study design. Non-randomized experimental and quasi-experimental studies were evaluated using criteria derived from the JBI checklists for quasi-experimental studies (JBI, n.d.); observational, cross-sectional, or correlational studies were evaluated using JBI criteria for cross-sectional studies; descriptive-normative or stimulus-validation studies were evaluated using descriptive criteria compatible with JBI; and the randomized controlled trial by Lee et al. (2022) was evaluated using RoB 2 (Sterne et al., 2019)/JBI-RCT criteria. When the information required to assess a specific methodological item was not explicitly reported in the codebook, extraction forms, or original study report, the item was coded as unclear. Missing information was not inferred, completed, or assumed. This conservative coding strategy was adopted to minimize reviewer assumptions, avoid overinterpretation, and preserve transparency in the appraisal process. Given the methodological diversity of the included studies, the assessment of the methodological quality was conducted using design-specific criteria and was intended to inform, rather than determine, the synthesis. Accordingly, methodological appraisal was not used as an exclusion criterion, but as a means of weighting the confidence placed in the findings across domains. Evidence derived from studies with small samples, limited reporting, *post hoc* grouping, lack of control conditions, or unclear methodological procedures was interpreted with greater caution, whereas findings from studies with larger samples, clearly specified designs, objective outcome measures, and well-described procedures were given greater interpretative weight in the narrative synthesis.

Narrative synthesis was selected as the main synthesis strategy because of the heterogeneity of the corpus. The synthesis was structured by cognitive domain, with particular attention to convergent findings, contradictory results, and methodological limitations.

## 3 Results

### 3.1 Study selection

The final corpus of the scoping review comprised 55 studies investigating the relationship between magic and human cognition. Attention and misdirection constituted the largest domain (*n* = 17; 30.91%), followed by high-level cognition (*n* = 15; 27.27%), epistemic emotion and memory (*n* = 10; 18.18%), visual perception (*n* = 8; 14.55%), and neural correlates (*n* = 5; 9.09%).

**Figure 2:**
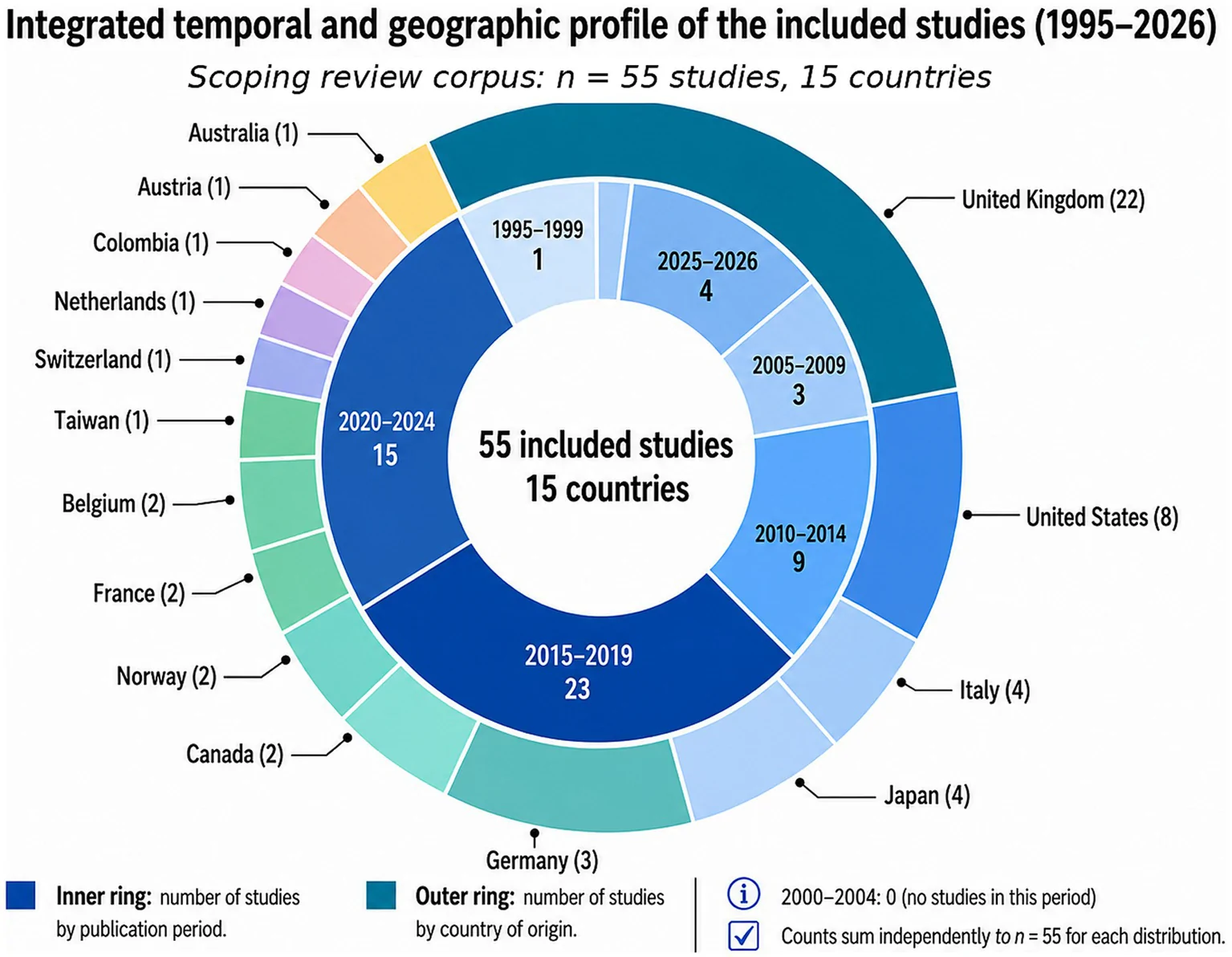
Integrated temporal and geographic profile of the included studies (1995–2026).

The temporal and geographic profile of the included studies is summarized in a single integrated figure (Figure 2), in which an inner ring depicts the distribution of studies by publication period and an outer ring depicts their distribution by country of origin, each expressed as an independent count that sums to the full corpus (*n* = 55). From a temporal perspective, scientific production followed an uneven but cumulative trajectory, with a marked concentration of publications from the 2010s onward and the largest single period corresponding to 2015–2019 (*n* = 23); within this span, 2016 stood out as the single most productive year, after which output remained comparatively stable. Geographically, the corpus was concentrated in a small number of countries: the United Kingdom accounted for nearly two out of every five included studies (*n* = 22; 40.0%), followed by the United States (*n* = 8; 14.5%), with the remaining studies distributed across Italy and Japan (*n* = 4 each), Germany (*n* = 3), Canada, Norway, France, and Belgium (*n* = 2 each), and six countries contributing a single study each (Taiwan, Switzerland, the Netherlands, Colombia, Austria, and Australia). This pattern is consistent with the concentration of several leading research groups in the science of magic and visual cognition within British institutions, and it indicates that, despite the field’s growing temporal consolidation, empirical research on magic and cognition remains geographically narrow—a pattern that constrains the generalizability of the reviewed findings.

### 3.2 Domain D1: attention and misdirection

The largest group of included studies focused on attention and misdirection (*n* = 17). These studies analyzed how magical effects direct, capture, divide, or inhibit spectators’ attention, as well as the mechanisms through which observers may fail to detect a critical event even when it occurs within the visual field. Most studies used magic tricks presented on video, although some incorporated live or more naturalistic conditions. The most frequent methodological approach was eye-tracking, usually combined with behavioral detection tasks, forced-choice responses, verbal reports, or post-trick explanations. Taken together, this domain provides the most consolidated empirical evidence within the corpus, with several studies converging on the idea that misdirection cannot be understood solely as a displacement of gaze, but rather as a dynamic interaction between overt attention, covert attention, expectation, timing, and social information. Despite the diversity of paradigms and measures employed, the studies grouped in this domain share the common conceptual core of examining how magical effects modulate the allocation of attentional resources and the conscious detection of critical events. From this perspective, the domain includes both studies focused directly on overt and covert attention, as well as work that extends the domain toward closely related phenomena, such as inattentional blindness and the implicit registration of undetected events.

An initial important contribution comes from studies examining the role of gaze cues in magical misdirection, a technique that consists of diverting the spectator’s attention. This line of research was first established by Kuhn et al. (2008a,b), who used a prerecorded magic trick to examine the factors underlying successful misdirection. Prior knowledge of the trick’s method significantly improved detection, and informed participants fixated closer to the critical event, suggesting closer monitoring once the method was known. Critically, participants who detected the critical event of the trick began orienting their eyes toward that location considerably earlier than those who missed it, demonstrating that attention can be allocated ahead of the current fixation point, and laying the empirical groundwork for subsequent closely related studies. Kuhn et al. (2009) showed that the magician’s gaze can influence spectators’ eye movements and reduce detection of the critical event. That is, the magician’s own gaze conditions spectators’ gaze. However, their findings also suggested that the overt direction of gaze was not sufficient to fully explain why spectators could miss the critical (hidden) event in a trick. Although the magician’s gaze reoriented spectators’ visual fixation, the study concluded that misdirection involves not only overt attention, but also covert attention, understood as the allocation of attentional resources without a corresponding shift in gaze.

This distinction was subsequently reinforced by Kuhn and Findlay (2010), who used a magic trick as an inattentional-blindness paradigm to examine the temporal relationship between eye movements and visual awareness. The magic trick consisted of making a lighter disappear while directing the spectator’s attention toward another point. Most people did not see the lighter fall. To ensure that this was genuine perception rather than a subsequent inference, the authors created a second version of the video in which the fall was digitally removed. Their findings showed that observers may fail to consciously perceive a visually salient event even when their eyes are directed toward, or near, the relevant area of the scene. This result challenged a simple gaze-based explanation of misdirection, since looking toward the critical region does not necessarily entail consciously detecting the secret action. The study therefore showed that magic does not deceive merely by redirecting the spectator’s gaze, but by reallocating attention.

If what determines the success of the illusion is attention rather than gaze alone, one might expect social cues, which are known for their capability to capture attention, to function as the misdirection mechanism par excellence. In this line, Cui et al. (2011) examined whether the visible presence of the magician’s face (i.e., a clear social cue) increased the effectiveness of a coin-vanishing illusion. Contrary to what might be expected, the presence of the face reduced, rather than increased, the credibility of the illusion. This finding is theoretically relevant because it shows that social information does not automatically enhance misdirection. Instead, its effect depends on how it integrates with the physical structure of the trick, the timing of the critical action, and the spectator’s expectations. The effectiveness of misdirection depends not only on the presence of social information, but on its functional relationship with the mechanics of the magical effect. A broadly compatible conclusion was later reported by Nguyen et al. (2026), who found that a magician’s spoken narrative, whether congruent or incongruent with the card movements, or absent altogether, had no reliable effect on participants’ ability to notice a fully visible identifying mark during a Three-Card Monte routine. The authors explicitly linked this null result to Cui et al. (2011), postulating that socially or verbally mediated misdirection cues may be redundant once the deceptive action itself, such as well-executed sleight-of-hand, is sufficiently robust on its own.

Another important line of research in this domain focused on inattentional blindness and perceptual load. Barnhart and Goldinger (2014) showed that prior knowledge of the general structure of an event can reduce inattentional blindness in a magic-based paradigm. This finding suggests that detection failures are not merely passive perceptual errors but depend in part on how observers organize the event and allocate their attentional resources. When spectators possess a more informative representation of the scene, they can allocate attention more efficiently and increase the probability of detecting the unexpected event. In this sense, magic-based paradigms allow inattentional blindness to be studied in situations that are more dynamic, structured, and meaningful than many conventional laboratory tasks.

More recent work has incorporated more subtle indicators of attentional allocation. Barnhart et al. (2019), for example, demonstrated that microsaccades reflect the dynamic deployment of covert attention during magical misdirection, providing an index that goes beyond traditional patterns of overt fixation. This line of evidence reinforces the idea that misdirection is not a single mechanism, but a complex attentional process involving multiple levels of visual, oculomotor, and cognitive control. A similar logic has also been extended to the knowledge that remains after a trick has concluded. In this sense, Kawakami and Miura (2017) compared whether information regarding the critical event of a trick could be better predicted by a direct measure of detection or by an indirect, unconscious measure (i.e., an implicit association test). The unconscious measure was a better predictor than the conscious report, suggesting that the perceptual failure subjectively experienced during misdirection does not necessarily entail a complete absence of cognitive registration of the critical event.

Extending the paradigm to a clinical population, Kuhn et al. (2010) compared individuals with autism spectrum disorder (ASD) and typically developing controls on the vanishing-ball illusion. Contrary to the prediction that reduced sensitivity to social cues would make the ASD group less susceptible to misdirection, participants with ASD were significantly more susceptible to the illusion. Eye-tracking data showed that both groups attended similarly to the magician’s face and eyes, but the ASD group was slower to initiate saccades toward the face and less likely to fixate on the ball at the critical moment, suggesting that the heightened susceptibility stemmed from subtle delays in the temporal allocation of attention rather than from avoidance of social cues. This finding therefore reinforces a central point of this domain: the efficacy of misdirection does not depend solely on exposure to social cues, but on the precise timing with which attention is deployed toward them.

Exploring a different attentional mechanism, Wiseman and Nakano (2016) examined spontaneous eye-blinking as a potential marker of attentional relaxation during the observation of a magic trick. Participants’ blinks were highly synchronized across viewers and tended to cluster around the magician’s secret actions rather than around the seemingly impossible effects. This finding suggests that blinking, and the associated momentary relaxation of attention it reflects, may constitute an additional, previously unrecognized channel through which magicians conceal critical actions from an attentive audience.

Overall, the evidence in this domain converges on three main conclusions. First, misdirection operates in part through covert attention and cannot be explained solely by where spectators are looking. Second, social cues have context-dependent effects and can facilitate or weaken the illusion depending on their timing and integration with the physical action. And third, magic-based paradigms provide a valuable avenue for studying attention dynamically, as they combine spatial orienting, temporal expectation, social information, and perceptual uncertainty within ecologically meaningful events. Important limitations remain, including the relatively small samples, the variability of the tricks and dependent variables, and the diversity of experimental paradigms. Despite these limitations, attention and misdirection constitute the most empirically consolidated domain in the reviewed literature and offer one of the strongest arguments for the use of magic as a methodological tool in cognitive science.

### 3.3 Domain D2: visual perception

The second domain included studies focused on visual perception (*n* = 8). Although not all studies are described individually in this synthesis, the studies grouped within this domain share a common thread: examining how magical effects exploit mechanisms of perceptual construction, visual prediction, completion of hidden information, and dynamic scene updating. The studies discussed below illustrate the main empirical patterns observed within this category.

In this regard, the so-called Vanishing Ball Illusion has been particularly relevant. In this trick, a magician repeatedly throws a ball into the air and, on the final throw, simulates throwing it again while keeping it hidden in their hand. Most observers perceive the ball as rising and disappearing, even though it never actually left the magician’s hand. Kuhn and Rensink (2016) showed that this illusion can occur without prior exposure to an actual throw, suggesting that long-term knowledge about object dynamics is sufficient to generate an illusory perceptual prediction, with a dissociation between conscious experience and oculomotor response. If the perceptual system can generate this illusion based solely on learned expectations, it is worth asking what other factors, beyond prior visual experience, might modulate its strength. In this regard, Begey et al. (2023) showed that congruent auditory information increases the probability of experiencing the visual illusion, although it does not amplify its intensity among those who already experience it. They tested this question using a modified version of the same illusion, replacing the ball with a coin, giving rise to the Vanishing Coin Illusion. The magician simulates throwing the coin into the air while keeping it hidden in the palm of the hand. The authors conducted two successive experiments. In the first one, they compared the presence versus absence of a sound during the simulated throw, and found that the mere presence of a sound already increased the success of the illusion (i.e., more participants perceived the coin as rising and disappearing). In the second experiment, they refined this question by comparing a sound congruent with the throw against an incongruent one. The results showed that the congruent sound increased the probability that a participant would experience the illusion, but did not intensify the experience among those who were already deceived regardless. In other words, auditory congruence helps more people fall for the illusion but does not make the illusion more convincing for those who were already deceived by the visual stimulus alone. A related earlier extension was provided by Tompkins et al. (2016), who developed the Phantom Vanish Trick to test whether misdirection could induce the perception of an entirely non-existent object, rather than merely the illusory disappearance of a real one. Using silent video stimuli to rule out verbal suggestion, they found that around one third of the tested participants reported perceiving an object that had never been presented, with the majority of these reports being congruent with objects shown in preceding trials. This finding reinforces the inferential account of magical illusions, showing that top-down expectations can be sufficient to generate a vivid perceptual experience even in the complete absence of corresponding sensory input.

While other sensory modalities modulate the impact of a trick, the visual system itself possesses inferential mechanisms that sustain magical illusions even without any external support. Ekroll et al. (2016) demonstrated that visual amodal completion of the invisible part of an object can generate measurable perceptual and bodily consequences, and Ekroll et al. (2018) showed that tricks based on amodal completion, understood as the visual system’s tendency to complete the hidden parts of an object, are resistant to explicit knowledge; even when the spectator knows the method, the perceptual illusion can persist. This resistance to explicit knowledge does not appear to be limited to amodal completion but also emerges in other effects based on distinct inferential mechanisms. This is precisely what Svalebjørg et al. (2020) showed, extending this finding to the illusion of absence, and demonstrating that discovering the secret did not significantly reduce the “magical” rating of the effect.

In a complementary line, Yao et al. (2019) examined change blindness, showing that an abrupt change in movement direction can induce detection failures even without the need for explicit occlusion. This result connects magical illusions with basic mechanisms of dynamic visual scene updating. The observer does not simply perceive an objective succession of stimuli but integrates movement within a perceptually plausible sequence. In other words, when a change is inserted within an apparent continuation of the sequence of actions, conscious experience may omit any relevant modification of the stimulus. This study therefore reinforces the idea that magic allows the study not only of disappearance or completion illusions, but also of the limits of visual perception in detecting dynamic changes within moving scenes.

Findings in this domain converge on the central conclusion that visual perception is not a passive record of sensory input, but an active inferential process in which prior expectations shape what is experienced. This principle holds consistently across distinct phenomena such as the completion of hidden parts of an object, the persistence of an illusion despite knowing its mechanism, and the modulation of its strength through information from another sensory modality. Magical illusions based on amodal completion and on perceptual persistence despite explicit knowledge of the method illustrate that these inferential mechanisms operate in an encapsulated manner, resistant to high-level cognitive intervention. This positions magic as a particularly suitable paradigm for testing models of predictive perception, as it allows for the experimental dissociation of what is perceived from what is known.

### 3.4 Domain D3: high-level cognition

Although the studies included in this domain (*n* = 15) are heterogeneous in their paradigms and variables, they share a common conceptual core: they use magic as an experimental context to study cognitive processes that go beyond immediate perception, such as problem solving, insight, mental fixation, explanation generation, decision-making without awareness, trick evaluation, perceptual-motor expertise, and mental imagery. From this perspective, the domain does not group studies by the superficial similarity of the tricks employed, but by the type of high-level cognitive operation that these tricks make it possible to investigate. Danek et al. (2014) proposed magic tricks as an ideal paradigm for studying insight during problem solving, in contrast to step-by-step analytic solutions. To this end, they presented participants with videos of magic tricks and asked them to try to discover, through repeated viewings, the secret method used by the magician. Subsequently, they classified each solution reached as achieved through insight or through analytic reasoning, based on participants’ own accounts of how they arrived at the conclusion. Their results showed a pattern that might seem counterintuitive at first glance: when a trick’s solution arrived through insight, it was more frequently correct and was also accompanied by greater subjective confidence, compared with solutions reached analytically.

If insight is associated with more accurate and confident solutions, one might ask what happens at the preceding stage, when the reasoner has not yet reached a solution and feels stuck. This is precisely what Pétervári and Danek (2020) examined. They asked participants to decipher the secret method behind a series of magic tricks and deliberately increased the likelihood of an impasse after the first viewing by presenting two implausible alternative solutions and informing participants that both were incorrect. After a second viewing, cues were provided to help them overcome this impasse. In doing so, the authors showed how eliminating readily available but incorrect solution candidates can guide participants into an impasse, and how subsequent cues can help them move beyond it. If such cues help overcome a deliberately induced block, one might ask what happens when the trick itself, without any experimental intervention, induces a mistaken idea that is difficult to abandon. The study by Thomas and Didierjean (2016) provides compelling evidence in this same line. They showed that magic tricks can generate mental fixation, as the spectator becomes anchored to an unlikely explanation of the effect, making it difficult to consider a more obvious solution. In their study, a single verbal exposure to a false and unlikely explanation of the trick was enough to prevent participants from later finding the real, much simpler solution (in their case, that all the cards used in the trick were identical). Even when participants were explicitly invited to search for an alternative explanation to the one already ruled out, most of them (80%) still failed to find it, instead assuming that the trick had no simpler solution.

If a single exposure can anchor some individuals so firmly to an incorrect explanation, the question remains whether this susceptibility depends not only on the trick itself, but also on individual differences in reasoning. Gronchi and Zemla (2021) showed precisely that cognitive style predicts the type of explanation generated for mental magic tricks, linking magic to the dual-process theory. In a related vein, Olson et al. (2015a) showed that a forcing technique—a procedure by which a magician steers an apparently free choice toward a predetermined option—can influence participants’ decisions without their awareness, leading them to generate retrospective explanations that feel genuine but do not reflect the actual processes underlying their choices. Subsequently, Olson et al. (2015b) used a vanishing trick to examine developmental changes in causal explanation. Younger children were more likely to interpret the apparent event literally or invoke supernatural causes, whereas older children generated increasingly plausible mechanistic explanations. Confidence, however, did not improve monotonically with explanatory sophistication. Rather, it decreased across childhood but increased again in adulthood, leaving both younger children and older adults relatively overconfident in inaccurate explanations. The study therefore illustrates how magic can dissociate the quality of causal explanations from metacognitive confidence across the lifespan. This capacity of misdirection paradigms to reveal biased self-knowledge was also examined directly by Ortega et al. (2018), who used four magic tricks exploiting inattentional and change blindness to study metacognitive biases in visual awareness. Participants who detected a trick’s method judged that other people would also be more likely to detect it and believed they had fixated the critical area more often than those who missed it. Their eye-tracking data, however, showed that most successful detections actually occurred through peripheral rather than foveal vision, directly contradicting participants’ own beliefs about how they had detected the method. This dissociation shows that successful detection does not necessarily provide accurate introspective access to the attentional processes that produced it.

Beyond these processes of explanation, belief, and decision-making, another set of studies within this domain focused on a different aspect of high-level cognition: the perceptual-motor expertise that magicians themselves develop as a result of their expert practice. Studies on expertise showed that magicians outperform non-magicians at detecting pantomimed grasps (i.e., simulated grasping movements directed toward an absent or imagined object; Quarona et al., 2020; Garcia-Pelegrin et al., 2022). Other studies reported differences in the bodily representation of the hand (Cocchini et al., 2018) and in the execution of real versus pantomimed actions (Cavina-Pratesi et al., 2011). Rinsma et al. (2017), however, showed that pantomimed grasping is controlled by the ventral system in both magicians and non-magicians, suggesting that magical expertise modifies certain aspects of visuomotor processing and motor execution, but does not eliminate the general constraints of the human visuomotor system.

Finally, Ekroll et al. (2024) showed that certain tricks based on mental imagery can be adapted to non-visual modalities with comparable results. The authors started from the idea that some topological magic tricks, which use flexible materials such as ropes, cloths, or rubber bands, do not depend on visual perception per se, but on the limitations and expectations of mental imagery. To test this idea, they selected three tricks presumably based on this mechanism and compared their presentation in the original visual format with a version adapted for non-visual presentation. One of the tricks used involved a rope that appeared to be cut in half, when in fact only a small fragment of one of its ends was actually cut, an effect that relies on amodal completion, that is, on the cognitive system’s tendency to assume that the rope remains continuous despite the manipulation. The results showed that the non-visual version of these tricks maintained a magical effect comparable to that of the original visual version, supporting the idea that what is deceived is not the eye, but the mental simulation constructed regarding how the object should behave.

Altogether, the studies included in this category show that magicians’ expertise modulates specific aspects of visuomotor processing and action detection, such as the recognition of pantomimed grasps or the bodily representation of the hand, without altering the general constraints of the human visuomotor system, which operates similarly in magicians and non-magicians. Beyond this perceptual-motor expertise, magic emerges as a useful paradigm for studying the limits of metacognition, introspection, and the subjective sense of control.

### 3.5 Domain D4: epistemic emotion and memory

The fourth domain included studies focused on epistemic emotion and memory (*n* = 10). Epistemic emotions are affective responses elicited by the pursuit, acquisition, or revision of knowledge, including states such as curiosity, interest, and surprise. Ozono et al. (2021) developed MagicCATs, a standardized collection of 166 videos designed to induce curiosity, surprise, and interest, with normative data. The videos include a wide variety of materials, such as cards, cups and balls, rings, and coins, and incorporate different types of effects, including misdirection, illusion, and forcing. The information presented is predominantly non-verbal, which facilitates intuitive understanding of the content and evokes curiosity, surprise, or interest regardless of the observer’s language, educational level, or cultural background. The rating data collected by the authors themselves confirm that the tricks included in the collection elicit a sufficient variety of epistemic emotions, with adequate variability across stimuli, supporting their psychometric properties for use in psychological experiments.

Meliss et al. (2024) published the first open neuroimaging dataset using validated tricks and an incidental-memory assessment. In their study, 50 participants watched 36 magic tricks recorded and edited specifically for functional magnetic resonance imaging experiments, while their brain activity was recorded before, during, and after viewing. The design included curiosity ratings for each trick, and an incidental-memory test on the content of the tricks administered 1 week later, in addition to individual working-memory measures and other variables related to motivated learning. The authors made both the behavioral and neuroimaging data publicly available, validated through variance-decomposition and functional-connectivity analyses. Separately, Marascia et al. (2026) extended the validation of the MagicCATs stimuli to an Italian cultural context, providing normative data on curiosity, interest, and surprise from a large set of participants who rated the videos, and Padulo et al. (2026), drawing on a related Italian sample, incorporated machine learning showing that the co-occurrence of curiosity, interest, and surprise depends on the specific interaction between the person and the particular trick, rather than on a fixed pattern applicable to any combination of spectator and stimulus.

From a historical standpoint, empirical interest in the intersection of magic and memory can be traced back to Wiseman and Morris (1995), who demonstrated that non-believers in paranormal phenomena better remembered aspects relevant to detecting deception, providing an early indication that the relationship between belief and memory in response to magical stimuli is not neutral. More than two decades later, Moss et al. (2017) extended research on magic and memory to a different applied context, showing that magic can reduce engagement with subsequent material when the secret is not revealed (see also Wiseman et al., 2020, for a replication using a similar approach in an educational context). Research subsequently broadened to examine the emotional and experiential responses elicited by magic. Lewry et al. (2021) found that tricks violating physical principles acquired earlier in development were rated as more surprising. In a separate applied line, Bagienski et al. (2022) reported subjective improvements in students’ wellbeing after learning magic tricks, comparable to those observed following a mindfulness intervention. In a related line of research, Bagienski and Kuhn (2023) showed that enjoyment of a magic performance increased as the trick became progressively more impossible, but that this relationship was nonlinear: enjoyment plateaued after the first impossible moment, whereas perceived impossibility continued to increase. This pattern suggests that unexpected surprise, rather than impossibility itself, may drive the enjoyment of magic.

Taken together, these studies show that magic tricks are effective stimuli for eliciting epistemic emotions such as curiosity, surprise, and interest, making them attractive tools for both research and applied contexts. However, the findings in this domain indicate that greater engagement, that is, the degree of attentional and emotional involvement of the observer with the stimulus, does not necessarily entail a direct improvement in memory for the associated information. Although subjective absorption in a trick may be high, this does not automatically translate into better factual recall of related content, and in some cases, magic may even reduce subsequent engagement if the trick’s secret is not revealed. These results suggest that epistemic emotion and memory are partially dissociable processes, and that the effectiveness of magic as an educational or therapeutic tool depends on how the relationship between the immediate emotional experience and the subsequent consolidation of information is managed.

### 3.6 Neural correlates of the magical experience

Unlike the four preceding categories, which are defined primarily by the cognitive process examined, D5 was defined by its level of analysis and included studies reporting neural measures during magic observation or neurocognitive outcomes following magic-based interventions. Specifically, this category includes studies (*n* = 5) that examined neurocognitive evidence related to the magical experience. The processes examined in these studies, such as conflict detection, expectation violation, predictive processing, and neurocognitive change, may contribute to several of the cognitive phenomena described in the preceding sections.

Parris et al. (2009), in the first fMRI study on magic, identified activation of the left DLPFC (BA 6, 8, 46) and the left dorsal ACC (BA 32) during the observation of tricks, with DLPFC activation being specifically greater for tricks than for surprising events, suggesting a role in the conflict between perceptual experience and causal knowledge. Later, Danek et al. (2015) used fMRI to examine neural responses while participants watched video clips of magic tricks and matched control events. The magic clips violated the expected causal relationship between an action and its outcome, whereas the control clips preserved a plausible action–outcome sequence. This design allowed the authors to separate the neural activity associated with the immediate moment of expectation violation from that associated with the subsequent attempt to understand the trick. They identified two response patterns: a transient response at the moment of expectation violation (involving the left inferior frontal gyrus, the anterior insula, and the occipital cortex), and a more sustained response during trick resolution (characterized by bilateral activation of the caudate nucleus). Additionally, Danek et al. (2014) investigated the neural activity of the professional magician, and revealed a qualitatively different activation pattern. Whereas naïve observers showed the frontal and subcortical pattern described above, the magician showed activation primarily in the bilateral supramarginal gyrus, without the frontal pattern associated with expectation-violation detection. This result supports the hypothesis that, by knowing the trick’s method, the magician does not experience the event as a causal violation, suggesting that expert knowledge of the method can modulate the neural response to expectation violation. In a related EEG study, Caffaratti et al. (2016) recorded event-related potentials (ERPs) while participants watched a magician perform a ball-and-cup manoeuvre and predicted the ball’s final location. The electrophysiological response varied as a function of participants’ expectations about the outcome, indicating that the trick engaged anticipatory neural processes before its resolution. This evidence suggests that observing magical effects involves not only a subjective response of surprise, but also anticipatory processes related to expectations about the event’s outcome.

Along the same line, Plikat et al. (2025) used magic tricks in an fMRI experiment and revealed a hierarchical organization of neural responses to naturalistic violations of expectation: the dorsal ACC and precuneus were associated with generic surprise responses, whereas specific sensory areas were recruited depending on event type. Furthermore, knowledge of the method did not eliminate neural surprise activity, which contrasts with the finding by Danek et al. (2015) in the expert magician, whose frontal activation associated with expectation violation was absent. This discrepancy may reflect differences between the deep, automatized knowledge that a professional magician possesses about their own repertoire of tricks and the explicit but recently acquired knowledge of a participant who has been told the method behind someone else’s trick.

Finally, Lee et al. (2022) extended this category to an intervention context, reporting changes in cognitive function and neurocognitive performance after a magic-based intervention program in older adults with mild cognitive impairment. Although this study differs from the observational neuroimaging and electrophysiological studies described above in that EEG evidence was used not to explore magic processing but rather the outcome of a magic-based training, it was included in this category because its primary contribution concerned neurocognitive outcomes associated with a magic-based intervention.

In conclusion, this category shows that the magical experience recruits general neural networks involved in conflict detection, expectation violation, prediction, and updating, rather than a substrate exclusive to magic. Likewise, the available evidence suggests that knowledge of the method can modulate this response, although its effects may depend on the type and depth of that knowledge. This is, however, the category with the fewest studies and the greatest methodological heterogeneity in the entire corpus, which limits the scope of these conclusions. Nevertheless, it provides preliminary neurocognitive evidence that complements the behavioral findings described in the preceding categories.

### 3.7 Preliminary methodological appraisal and synthesis strategy

A preliminary methodological appraisal was conducted using design-specific criteria informed by the relevant JBI checklists and, for the randomized trial, RoB 2. Given the heterogeneity of the included designs, the appraisal was descriptive and was used to contextualize, rather than formally grade, the evidence. All the 55 records in the corpus were evaluated using methodological quality or risk-of-bias tools adapted to study design. Overall, 14 studies were classified as having relatively higher methodological quality, 34 as having moderate methodological concerns, and 7 as having substantial methodological concerns. The relatively higher-quality category was concentrated mainly among normative studies or studies with large samples, clear experimental designs, and well-defined measurement procedures. Moderate quality was the predominant category, generally because of acceptable samples but incomplete information on randomization, absence of preregistration, stimulus heterogeneity, or limitations inherent to online designs. Studies classified as having substantial methodological concerns were characterized mainly by very small samples, *post hoc* groups, lack of adequate control, limited methodological information, or observational designs with reduced capacity to establish causal inferences (see Table 2).

**Table 2:** Preliminary synthesis of methodological quality by cognitive domain.

Domain	Higher relative methodological quality	Moderate methodological concerns	Substantial methodological concerns
D1 Attention and misdirection	3	9	5
D2 Visual perception	1	7	0
D3 High-level cognition	4	10	1
D4 Epistemic emotion and memory	6	4	0
D5 Neural correlates	0	4	1
Total	14	34	7

These results should be considered descriptive and do not imply exclusion of studies from the synthesis. Instead, they inform the overall level of confidence attributed to the findings within each domain. Domains characterized by a higher proportion of studies with small samples, post hoc designs, or incomplete methodological information are discussed with corresponding caution, whereas domains with a stronger concentration of large-sample, clearly controlled studies support comparatively more confident interpretive statements.

## 4 Discussion

This scoping review mapped and synthesized 55 empirical studies on the relationship between illusionism and human cognitive processes, organized into four cognitive domains and a category of neural correlates. The results support the value of magic as a methodological tool for the study of human cognition, although the degree of empirical consolidation varies across domains. The attention and misdirection domain offers the most consistent evidence; the dissociation between ocular fixation and covert attention has been documented independently in multiple laboratories. In visual perception, studies on amodal completion and the illusion of absence show that certain illusions are cognitively impenetrable. In high-level cognition, magic-based paradigms show that observers can become fixed on incorrect causal explanations, remain unaware of the factors guiding apparently free choices, and misidentify the attentional processes that enabled them to detect a trick. These findings reveal systematic limits in metacognitive access and in the subjective sense of agency over one’s own decisions. In epistemic emotion and memory, the creation of standardized stimuli such as MagicCATs represents a notable methodological advance. With regard to the neural correlates, the available studies suggest the recurrent involvement of frontal and cingulate regions, including DLPFC, ACC, and IFG, in the processing of the experience of impossibility, although the small number of studies prevents the establishment of a definitive neural model.

The findings are broadly consistent with the foundational theoretical proposals of the field, several of which were not included in the review corpus because of their conceptual rather than empirical nature, but nevertheless provide an important interpretive framework for the present synthesis. Kuhn et al. (2008a), for example, argued that magic can offer unique insight into attention, perception, and decision-making because it preserves a degree of ecological validity that is often difficult to achieve in conventional laboratory paradigms. The empirical findings reviewed here support this general claim by showing that magic-based paradigms can reveal how attentional, perceptual, predictive, and decision-related processes operate in contexts of uncertainty and expectation violation.

At the same time, the evidence also suggests that some of the field’s initial conceptual distinctions need to be refined. In particular, the traditional distinction between physical and psychological misdirection has proven useful as a starting point, but it does not fully capture the temporal and predictive dimensions of misdirection observed across the studies. A more comprehensive account should therefore consider not only where attention is directed, but also when critical information is presented, how expectations are generated, and how prediction errors are resolved.

This need for conceptual refinement is also consistent with the methodological caution introduced by Cole and Millett (2023). Their findings suggest that principles derived from magical practice should not be assumed to constitute valid psychological mechanisms without prior empirical verification. In other words, the fact that a magician claims that a technique works because it exploits a particular psychological mechanism does not guarantee that this is the mechanism actually operating. Such claims must be tested experimentally before being incorporated as scientific evidence.

The findings here reported have implications for at least three areas of cognitive psychology. First, for models of attention, since the evidence reinforces the importance of distinguishing between overt and covert attention and supports the idea that perceptual awareness does not depend solely on gaze direction. Second, for models of predictive perception, since the resistance to explicit knowledge documented in visual perception (D2) and neural correlates is consistent with frameworks that posit a hierarchy of relatively encapsulated representations. And third, for models of reasoning and metacognition, considering that the D3 corpus shows that magic can produce systematic failures of introspection that go beyond those studied in classical choice-blindness or confabulation paradigms.

In the educational domain, the studies grouped under D4 suggest that magic tricks can increase engagement and curiosity, although these effects do not necessarily translate into improved recall. This distinction is important because the reviewed evidence indicates that the educational and cognitive value of magic may depend, at least in part, on whether the trick remains merely an unexplained illusion or becomes an object of explicit analysis. In this regard, tricks in which the secret is revealed appear to be more effective than those in which the method is permanently concealed, because revealing the underlying mechanism allows participants to reconstruct the causal structure of the effect, identify the discrepancy between perception and reality, and engage more actively with the cognitive processes involved. This distinction is particularly relevant for applied contexts, where magic is not used only to elicit surprise, but also as a structured activity that can promote learning, reflection, and cognitive engagement. In the clinical domain, the study by Lee et al. (2022) is particularly relevant. They found that a magic-based intervention improved general cognitive function, as measured by MoCA scores, and attentional and inhibitory performance, as reflected in Flanker-task accuracy, reaction times, and P3 amplitudes, in older adults with mild cognitive impairment. These findings are consistent with potential improvements in executive functioning. The authors discussed DLPFC involvement as a possible mechanism, although DLPFC activity was not directly measured. This evidence remains preliminary and should be interpreted with caution. Finally, the development and standardization of stimulus sets such as MagicCATs represents an important methodological step for the field, as it facilitates comparability across studies, reduces variability in the materials used, and allows future research to test more precisely which features of magical effects are responsible for specific cognitive outcomes.

The strengths of this review include the systematic search of three databases with no date restriction, complemented by a retrospective complementary search in APA PsycINFO, the exhaustive verification of the PRISMA-ScR flow, and the 35-field extraction protocol applied consistently to the 55 studies. The theoretically motivated organization of the evidence into four cognitive domains and one category of neural correlates, together with the inclusion of a preliminary methodological appraisal adapted to each study design, directly addresses the two overarching questions of the scoping review. Nevertheless, the methodological quality assessment was intended to be preliminary and descriptive rather than a formal risk-of-bias evaluation. Given the substantial heterogeneity of the study designs included in the corpus, direct comparisons across studies were not considered appropriate. Furthermore, all title-and-abstract screening, full-text assessment, and data extraction were initially conducted by one reviewer. A second reviewer independently verified the eligibility of all 55 included studies by examining their titles, abstracts, and full texts. Agreement regarding eligibility was 100%, and no disagreements required resolution. The second reviewer also independently verified the extracted information for the 55 included studies.

The analyzed corpus allows several priority directions for future research to be identified. The first concerns sample diversity. Most included studies originated in the United Kingdom and the United States and used adult university participants without specific clinical characteristics. It would be especially relevant to explore whether the effects of misdirection, the cognitive impenetrability of certain illusions, or the emotional response to impossible events are maintained in populations with different belief systems, different prior experiences with magic, or different patterns of cognitive development. Cross-cultural studies and studies incorporating diverse clinical or educational samples represent a particularly promising direction.

The second future direction concerns methodological rigor. The median sample size of the corpus is 50 participants. Considering that almost half of the studies worked with fewer than 50 participants, and that many studies lack a formal *a priori* power analysis, future research should incorporate preregistered designs, proper power calculations, and, where possible, direct replication studies of the most relevant effects. The creation of standardized stimulus databases such as MagicCATs represents an important step in this direction, and their systematic use in new studies would facilitate comparability across laboratories and cultures.

Another, less explored future direction concerns clinical and educational applications. The trial by Lee et al. (2022) suggests that magic-based interventions may have value beyond the laboratory as a resource in cognitive rehabilitation, particularly in older adults with mild cognitive impairment. However, the available evidence remains scarce and methodologically heterogeneous. Future studies with controlled designs, longitudinal follow-up, and clearly defined outcome measures would make it possible to assess more rigorously the applied potential of magic as a cognitive and therapeutic resource. Nevertheless, these findings open promising avenues for future clinical trials targeting specific neurodevelopmental conditions. For instance, structured cognitive training programs based on learning magic tricks, such as the MAGNITIVE intervention, have recently begun to show potential in community settings for children with attention-deficit/hyperactivity disorder, suggesting practical benefits in sustained attention and mental flexibility (Bonete et al., 2021). Such studies point to an applied value of magic that exceeds purely theoretical interest and justify a specific research line on its use as a therapeutic resource.

## 5 Conclusion

This scoping review supports the potential of magic as a valid and fruitful methodological tool for cognitive science, although its degree of consolidation varies according to the synthesis category analyzed. The available evidence, although heterogeneous in design and quality, converges in showing that magic tricks make it possible to study fundamental cognitive processes, including covert attention, perceptual prediction, causal reasoning, metacognition, and epistemic emotion, under conditions that conventional paradigms can hardly replicate.

The strongest findings come from the attention and misdirection domain. The dissociation between overt and covert attention, independently documented in multiple laboratories and with different paradigms, represents one of the most robust contributions of magic-based research to cognitive psychology. Thus, magical effects make it possible to study this dissociation under natural observation conditions that classical laboratory paradigms can hardly reproduce. Beyond attention, the review shows that certain magical illusions are cognitively impenetrable, and that explicit knowledge of the method does not necessarily eliminate the perceptual experience. The available evidence from the category exploring the neural signature of magic also suggests that expectation violation and the experience of impossibility recruit frontal and cingulate regions involved in conflict detection, prediction, and updating, although the small number and heterogeneity of studies prevent strong conclusions. Taken together, these findings have direct implications for predictive-processing models and for understanding the relationship between conscious knowledge, perceptual representation, and neural responses to impossible events.

The field has matured markedly since its initial programmatic formulations. It now has established and replicated paradigms, standardized stimulus databases, open datasets, and an active research community that has succeeded in turning the art of deception into a rigorous window onto the mechanisms of human cognition. Although magic may initially appear to be a complex, artisanal, or experimentally challenging phenomenon, the reviewed evidence shows that its principles can be operationalized, systematized, and used rigorously in scientific contexts. In this sense, magic is not merely an object of scientific study. It can also serve as a methodological instrument for investigating cognitive processes that are difficult to examine under ecologically valid conditions.

## Data Availability

The original contributions presented in the study are included in the article/Supplementary material, further inquiries can be directed to the corresponding author.
