# Supplementary material for: Magic and cognition: a scoping review of empirical evidence on cognitive and neural processes
Source: Front Psychol. 2026 Sep 3;17:1921905. doi: 10.3389/fpsyg.2026.1921905 (PMC13581636; doi:10.3389/fpsyg.2026.1921905)
Supplement: Supplementary file 1 [file Table_1.PDF]

Supplementary Material

Supplementary Table S1. Complete search strategies by database

The systematic literature search was initially conducted on 28 May 2026 across three electronic databases: Web of Science Core Collection (Clarivate Analytics), Scopus (Elsevier), and PubMed/MEDLINE (NLM/NCBI). An additional retrospective search was conducted on 15 July 2026 in APA PsycINFO via APA PsycNet, using the same conceptual boolean search strategy adapted to the platform, as recommended by an anonymous reviewer. No date restrictions were applied. The same conceptual search string was adapted to the field-tagging syntax of each platform. No post-search filters were applied at the database level; all filtering was carried out during the subsequent screening phases in Rayyan.ai.

<table>
<tr><th>Database</th><th>Platform</th><th>Filters</th><th>Results (n)</th><th>Date</th></tr>
<tr><td>Web of Science</td><td>Clarivate Analytics</td><td>None</td><td>442</td><td>28 May 2026</td></tr>
<tr><td colspan="5">Search string: TS=(("magic trick*" OR illusionism OR "sleight of hand" OR misdirection) AND (cognit* OR attention OR perception OR awareness OR memory OR "executive function*" OR neuro* OR brain) AND (experiment* OR "eye tracking" OR "eye-tracking" OR "reaction time" OR electroencephalography OR EEG OR fMRI OR "event-related potential*" OR ERP OR NIRS OR "near infrared spectroscopy"))</td></tr>
<tr><td>Scopus</td><td>Elsevier</td><td>None</td><td>483</td><td>28 May 2026</td></tr>
<tr><td colspan="5">Search string: TITLE-ABS-KEY((("magic trick*" OR illusionism OR "sleight of hand" OR misdirection) AND (cognit* OR attention OR perception OR awareness OR memory OR "executive function*" OR neuro* OR brain) AND (experiment* OR "eye tracking" OR "eye-tracking" OR "reaction time" OR electroencephalography OR EEG OR fMRI OR "event-related potential*" OR ERP OR NIRS OR "near infrared spectroscopy")))</td></tr>
<tr><td>PubMed / MEDLINE</td><td>NLM / NCBI</td><td>None</td><td>610</td><td>28 May 2026</td></tr>
<tr><td colspan="5">Search string: (("magic trick*" OR illusionism OR "sleight of hand" OR misdirection) AND (cognit* OR attention OR perception OR awareness OR memory OR "executive function*" OR neuro* OR brain) AND (experiment* OR "eye tracking" OR "eye-tracking" OR "reaction time" OR electroencephalography OR EEG OR fMRI OR "event-related potential*" OR ERP OR NIRS OR "near infrared spectroscopy"))</td></tr>
<tr><td>APA PsycInfo</td><td>APA PsycNet</td><td>None</td><td>98</td><td>15 July 2026</td></tr>
<tr><td colspan="5">Search string: (("magic trick*" OR illusionism OR "sleight of hand" OR misdirection) AND (cognit* OR attention OR perception OR awareness OR memory OR "executive function*" OR neuro* OR brain) AND (experiment* OR "eye tracking" OR "eye-tracking" OR "reaction time" OR electroencephalography OR EEG OR fMRI OR "event-related potential*" OR ERP OR NIRS OR "near infrared spectroscopy"))</td></tr>
</table>

Note. The search string was structured around three conceptual blocks connected by AND operators: (1) terms related to the magical stimulus or technique; (2) terms related to cognitive, perceptual, neural, or neuroscientific processes; and (3) terms related to empirical methodology or measurement. Truncation (*) was used to capture morphological variants. No language or date restrictions were applied. Field tags were adapted to each platform's syntax: TS=( ) for Web of Science, TITLE-ABS-KEY( ) for Scopus, free-text search for PubMed/MEDLINE, and Any Field search in APA PsycNet for APA PsycINFO.
