# Supplementary material for: Magic and cognition: a scoping review of empirical evidence on cognitive and neural processes
Source: Front Psychol. 2026 Sep 3;17:1921905. doi: 10.3389/fpsyg.2026.1921905 (PMC13581636; doi:10.3389/fpsyg.2026.1921905)
Supplement: Supplementary file 2 [file Table_2.PDF]

Supplementary Material

Supplementary Table S2. Data extraction codebook

This supplementary table describes the structure of the data extraction codebook used in the scoping review. The extraction spreadsheet contained one study-identification column (ID) and 35 extraction fields organized across five thematic blocks: bibliographic information (fields 1–7), methodological and design characteristics (fields 8–11), sample information (fields 12–16), features of the magical stimulus and cognitive outcomes (fields 17–25), and methodological appraisal and administrative metadata (fields 26–35). The ID column is not counted among the 35 extraction fields.

Supplementary Table S2. Codebook fields used for data extraction.

No.	Field name	Description
1	Extraction_status	Status of the extraction process and verification level for the record.
2	Authors	Authors of the study as reported in the source record or full text.
3	Year	Year of publication.
4	Country	Country or countries associated with the study, based on institutional affiliation of the authors.
5	Journal	Journal or publication outlet, including volume, issue and page or article information when available.
6	DOI	Digital Object Identifier, when available; coded as unavailable when no DOI could be verified.
7	APA7_reference	Full bibliographic reference formatted according to APA 7 style.
8	Study_objective	Main aim, research question or purpose of the study.
9	Design	Study design, including experimental, quasi-experimental, observational, normative, cross-sectional, neuroimaging or mixed design features.
10	Domain	Primary cognitive domain assigned for synthesis: attention and misdirection, visual perception, high-level cognition, epistemic emotion and memory, and neural correlate.

No.	Field name	Description
11	Publication_type	Type of publication, such as original empirical article, normative dataset, randomized trial, or theoretical/contextual record when applicable.
12	Total_N	Total sample size and, when available, sample size by experiment, group or subsample.
13	Age	Age information reported for the sample, including range, mean and/or standard deviation when available.
14	Sex_gender	Sex or gender distribution reported for the sample.
15	Population_type	Population type, such as adults, children, university students, clinical participants, magicians, non-magicians, or other specific groups.
16	Data_collection_context	Context in which data were collected, such as laboratory, classroom, online setting, live performance or neuroimaging environment.
17	Magical_phenomenon	Magical phenomenon, effect or technique used in the study.
18	Stimulus_task	Stimulus, task, procedure or paradigm used to expose participants to the magical event.
19	Comparison_condition	Control condition, comparison group or within-subject contrast, when present.
20	Cognitive_variable	Main cognitive, emotional, perceptual, mnemonic, metacognitive or neural process assessed.
21	Instrument_task	Instrument, behavioral task, scale, questionnaire, eye-tracking procedure, neuroimaging protocol or other measurement tool used.
22	Measure_type	Type of measure, such as behavioral, self-report, oculometric, psychophysiological, electrophysiological or neuroimaging.
23	Psychometric_information	Psychometric information or validation evidence for the instruments, when reported.
24	Main_result	Main finding or outcome extracted from the study.
25	Effect_size	Effect size, statistical magnitude or relevant inferential statistic reported by the authors, when available.
26	Consistency_with_previous_literature	Brief note on whether the findings are consistent with, extend or contradict prior literature.

No.	Field name	Description
27	Risk_of_bias_observation	Methodological-quality or risk-of-bias observation derived from the adapted appraisal criteria.
28	Reported_limitations	Limitations explicitly reported by the study authors or evident from the study report.
29	Funding	Funding information reported in the article.
30	Conflicts_of_interest	Conflict-of-interest statement reported in the article.
31	Included_in_qualitative_synthesis	Indicator of whether the record was included in the qualitative/narrative synthesis.
32	Potential_meta_analysis_inclusion	Assessment of whether the record could potentially contribute to a meta-analysis, given its design and outcome reporting.
33	Extractor_methodological_notes	Additional methodological notes recorded by the extractor to support interpretation and traceability.
34	Extraction_date	Date on which the extraction was completed or last updated.
35	Extractor	Person responsible for data extraction or verification.

Note. The complete extraction matrix (55 studies × 35 fields) is available as a separate spreadsheet file upon request. When information required to complete a field was not explicitly reported in the study, the field was coded as 'Not reported' or 'Unclear', following the conservative coding strategy described in the Methods section of the manuscript.
