# Supplementary material for: Magic and cognition: a scoping review of empirical evidence on cognitive and neural processes
Source: Front Psychol. 2026 Sep 3;17:1921905. doi: 10.3389/fpsyg.2026.1921905 (PMC13581636; doi:10.3389/fpsyg.2026.1921905)
Supplement: Supplementary file 3 [file Table_3.PDF]

Supplementary Material

Supplementary Table S3. Studies excluded at full-text assessment (n = 100)

The following table lists the 100 records assessed in full text and subsequently excluded, together with the assigned exclusion code. Records are ordered by exclusion code and then alphabetically by first author within each code.

Exclusion codes: E1 = Non-eligible population (n = 1); **E2** = Non-relevant phenomenon (n = 30); **E3** = No objective cognitive measure (n = 19); **E4** = Study without original empirical data (n = 47); **E5** = Non-eligible publication type (n = 2); **E6** = Full text not assessable in a language accessible to the reviewers (n = 1).

No.	First author	Year	Title	Exclusion code
1	**Garcia-Pelegrin**	2020	An unexpected audience: Experiments with magic effects might be informative about cognition in animals	**E1**
2	**Ager**	2022	The Scent of Ancient Magic	**E2**
3	**Ayres**	2022	The Sleight of Hand: Appearance and Disappearance of Things in Neo-Victorian Magic	**E2**
4	**Baron**	2014	Sleight of hand: Lawyer distress and the attribution of responsibility	**E2**
5	**Bartolini**	2023	Like a Magician Who Tricks the Eyes: Demonism, Epistemological Uncertainty, and Religious Heterodoxy in Seventeenth-Century Russia	**E2**
6	**Bear**	2008	From magician to metal brain: The embodiment of illusion in early European film theory	**E2**
7	**Belousova**	2021	The Magician and the Juggler in Nabokov's Aesthetic and Artistic Frame	**E2**
8	**Brown**	2020	Higher-order memory schema and consciousness experience	**E2**
9	**Chu**	2011	Magic Moore-Penrose inverses and philatelic magic squares with special emphasis on the Daniels-Zlobec magic square	**E2**
10	**Deacon**	2024	The magic in magician: Contributions of phonological dimensions of morphological awareness to children's reading development	**E2**
11	**Dung**	2025	A science of chimeras? The implications of illusionism for non-human consciousness research	**E2**

No.	First author	Year	Title	Exclusion code
12	**Friconnet**	2025	Phenomenal consciousness is alien to us: SETI and the Fermi paradox seen through the prism of illusionism and attention	**E2**
13	**Hoyet**	2012	Sleight of hand: Perception of finger motion from reduced marker sets	**E2**
14	**Humphrey**	2020	The Invention of Consciousness	**E2**
15	**Kuhn**	2025	What a ‘Landscape of Consciousness’ means for neurology and neuroscience	**E2**
16	**Lahoda**	2004	Alchemist and magician: Josef Sudek and Emil Filla	**E2**
17	**Marinsek**	2016	A Split-Brain Perspective on Illusionism	**E2**
18	**Marshall**	2010	Deception and Magic in Collaborative Interaction	**E2**
19	**McCormick**	2023	Unmasking Mathematical Fallacies in Operations Management: Two Mini-Case Studies Using Misdirection	**E2**
20	**Miller**	2023	The magic and misdirection of public service loan forgiveness in the United States	**E2**
21	**Moin**	2016	The magic of branding: The role of ‘pledge’, ‘turn’ and ‘prestige’ in fostering consumer trust in financial services	**E2**
22	**Morris**	2018	Interaction and learning in a humanoid robot magic performance	**E2**
23	**Nord**	2021	Culbert, Samuel: The Magician’s Work on Organization Change	**E2**
24	**Orthey**	2017	Strategy and Misdirection in Forced Choice Memory Performance Testing in Deception Detection	**E2**
25	**Palestini**	2020	Ephemeral graphics: Illusionism and representation in baroque machines	**E2**
26	**Pasquali**	2020	Your Eyes Never Lie: A Robot Magician Can Tell if You Are Lying	**E2**
27	**Pérez**	2020	Augmented illusionism: The influence of optical illusions through artworks with augmented reality	**E2**
28	**Sajid**	2025	‘Just stop doing everything for now!’: Understanding security attacks in remote collaborative mixed reality	**E2**
29	**Versluis**	2026	‘Magical relief’: The effectiveness of three stages of a video-based magic intervention on distress and pain in children	**E2**

No.	First author	Year	Title	Exclusion code
30	**Vul**	2007	Incubation benefits only after people have been misdirected	**E2**
31	**Wolpe**	1989	The derailment of behavior therapy: A tale of conceptual misdirection	**E2**
32	**Botella**	2026	Who Are the Magicians? Their Personality Traits	**E3**
33	**Clerici**	2021	Illusionist techniques as a complement to psychological support for children with cancer	**E3**
34	**Ezell**	2003	MAGIC WORKS (motivating activities geared-to instilling confidence — wonderful opportunities to raise kid's self-esteem)	**E3**
35	**Forster**	2025	The illusory perception of occluded space as empty depends on the occluded area	**E3**
36	**Frank**	2020	Shattering the Illusion of the Self-Earned Tip: The Effect of a Restaurant Magician on Co-Workers' Tips	**E3**
37	**Griffiths**	2015	Revealing ontological commitments by magic	**E3**
38	**Haritaipan**	2019	Impact of technical information in magic-based inspiration tools on novice designers	**E3**
39	**Haugland**	2021	Möbius Band Surprise: A Systematic Illusion in Imagery	**E3**
40	**Hines**	2019	A magic-themed upper limb intervention for children with unilateral cerebral palsy: The perspectives of parents	**E3**
41	**Kuhn**	2024	Experiencing the Improbable: How Does the Objective Probability of a Magic Trick Occurring Influence a Spectator's Experience?	**E3**
42	**Kuhn**	2023	Experiencing misinformation: The effect of pre-exposure warnings and debunking on psychic beliefs	**E3**
43	**Kumar**	2020	Attitude of higher secondary school teachers towards the use of magic tricks in the classroom	**E3**
44	**Li**	2023	Exploring the influence of magic performance on design creativity	**E3**
45	**Peretz**	2005	Magic trick: A behavioural strategy for the management of strong-willed children	**E3**
46	**Robieux**	2026	A Kind of Magic: Social Representations of Magic from Magicians and non-Magicians	**E3**
47	**Silvia**	2025	More than just a magic trick? Exploring an audience's supernatural attributions for magicians' performances	**E3**

No.	First author	Year	Title	Exclusion code
48	**Silvia**	2023	Who Hates Magic? Exploring the Loathing of Legerdemain	**E3**
49	**Wang**	2023	The effects of a magic-based intervention on self-esteem, depressive symptoms, and quality of life among community-dwelling older adults	**E3**
50	**Øhrn**	2019	A Perceptual Illusion of Empty Space Can Create a Perceptual Illusion of Levitation	**E3**
51	**Alexander**	2022	What the Neuroscience and Psychology of Magic Reveal about Misinformation	**E4**
52	**Bagienski**	2019	The crossroads of magic and wellbeing: A review of wellbeing-focused magic programs, empirical studies, and conceivable mechanisms	**E4**
53	**Beran**	2025	There's 'magic' in comparative cognition	**E4**
54	**Camí**	2020	On the cognitive bases of illusionism	**E4**
55	**Carbon**	2011	Using computer-animated magic tricks as a promising experimental paradigm for investigating perceptual processes	**E4**
56	**Cole**	2025	What Can Conjuring Tell Us About Cognition? The Future of the Science of Magic	**E4**
57	**Cole**	2020	Forcing the issue: Little psychological influence in a magician's paradigm	**E4**
58	**Curzon**	2017	Power of computational thinking: Games, magic and puzzles to help you become a computational thinker	**E4**
59	**Ekroll**	2017	The Other Side of Magic	**E4**
60	**Ekroll**	2019	Illusions of Imagery and Magical Experiences	**E4**
61	**Ekroll**	2016	Conjuring Deceptions: Fooling the Eye or Fooling the Mind?	**E4**
62	**Ekroll**	2013	Against better knowledge: The magical force of amodal volume completion	**E4**
63	**Eysenck**	2023	Fundamentals of Cognition	**E4**
64	**Ezell**	2026	Using Magic Tricks to Promote Social-Emotional Reciprocity and Peer Relationships Among Students with Autism Spectrum Disorder	**E4**
65	**Friesen**	2022	'It's all in your head': Magic and Misdirection in Medicine	**E4**

No.	First author	Year	Title	Exclusion code
66	**Gaitán**	2024	Illusionism, sleight of hand, ventriloquism and circus in Costa Rica (1941–1971)	**E4**
67	**Garcia-Pelegrin**	2024	Beyond the Tricks: The Science and Comparative Cognition of Magic	**E4**
68	**Heard**	2010	Do eye movements inform how a magic trick is understood?	**E4**
69	**Koch**	2012	In which you hear tales of scientist-magicians that make you look but not see	**E4**
70	**Kuhn**	2014	A psychologically-based taxonomy of misdirection	**E4**
71	**Kuhn**	2011	Misdirected by the gap: The relationship between inattentional blindness and attentional misdirection	**E4**
72	**Kuhn**	2015	Attention and Misdirection: How to Use Conjuring Experience to Study Attentional Processes	**E4**
73	**Kuhn**	2022	Misdirection — Magic, Psychology and its application	**E4**
74	**Kuhn**	2011	Misdirection — past, present, and the future	**E4**
75	**Kumari**	2018	Why Game Designers Should Study Magic	**E4**
76	**Lachapelle**	2008	From the stage to the laboratory: Magicians, psychologists, and the science of illusion	**E4**
77	**Macknik**	2009	Illusions and Illusionists: How to fool the brain with magic and other tricks	**E4**
78	**Macknik**	2008	Attention and awareness in stage magic: Turning tricks into research	**E4**
79	**Masters**	2021	Extended Goal Recognition: Lessons from Magic	**E4**
80	**Mohr**	2020	How Stage Magic Perpetuates Magical Beliefs	**E4**
81	**Most**	2010	What's 'inattentional' about inattentional blindness?	**E4**
82	**Nardi**	1984	Toward a social psychology of entertainment magic (conjuring)	**E4**
83	**Olson**	2021	Applying insights from magic to improve deception in research: The Swiss cheese model	**E4**
84	**Pailhès**	2021	Mind Control Tricks: Magicians' Forcing and Free Will	**E4**

No.	First author	Year	Title	Exclusion code
85	**Pailhès**	2020	A psychologically based taxonomy of magicians' forcing techniques: How magicians influence our choices, and how to use this to study free will	**E4**
86	**Polidoro**	2012	The magic in the brain: How conjuring works to deceive our minds	**E4**
87	**Pretorius**	2014	The survival of magician's magic: The dilemma of the influence of technology on the art of magician's magic	**E4**
88	**Putnik**	2024	From Black Magician to Proto-Feminist: The Reception of Cornelius Agrippa's Oeuvre Through the Centuries	**E4**
89	**Quiroga**	2016	Magic and cognitive neuroscience	**E4**
90	**Rensink**	2014	A framework for using magic to study the mind	**E4**
91	**Rolfe**	2016	Theatrical magic and the agenda to enchant the world	**E4**
92	**Stewart**	2016	Neuroscience and the magic of play therapy	**E4**
93	**Watt-Smith**	2013	Cardboard, Conjuring and 'A Very Curious Experiment'	**E4**
94	**Wiseman**	2020	Conjuring cognition: A review of educational magic-based interventions	**E4**
95	**Wu**	2023	A narrative review for clinical applications of magic	**E4**
96	**Zik**	2017	Giambattista Della Porta: A Magician or an Optician?	**E4**
97	**Kuhn**	2008	Towards a science of magic	**E4**
98	**Kuhn**	2010	Cognitive illusions: From magic to science	**E5**
99	**Tatler**	2007	Don't look now: The magic of misdirection	**E5**
100	**Petrov**	2022	Application of the inquiry-based learning approach in informatics education with a popular mathematical magic trick	**E6**

Note. Exclusion decisions were documented in Rayyan.ai at the time of full-text screening. Titles have been lightly standardized for presentation; full bibliographic details are available from the authors upon request.
