# Supplementary material for: Magic and cognition: a scoping review of empirical evidence on cognitive and neural processes
Source: Front Psychol. 2026 Sep 3;17:1921905. doi: 10.3389/fpsyg.2026.1921905 (PMC13581636; doi:10.3389/fpsyg.2026.1921905)
Supplement: Supplementary file 4 [file Table_4.PDF]

***Supplementary Material***

**Supplementary Table S4. Studies included in the scoping review (n = 55)**

The following table lists the 55 studies included in the qualitative synthesis. Records are ordered chronologically by year of publication and then alphabetically by first author within the same year.

**Synthesis categories: D1 = Attention and misdirection; D2 = Visual perception; D3 = High-level cognition; D4 = Epistemic emotion and memory; D5 = Neural correlates.**

| No. | First author | Year | Title | Synthesis category |
|---|---|---|---|---|
| 1 | **Wiseman** | 1995 | Wiseman, R. J., & Morris, R. L. (1995). Recalling pseudo-psychic demonstrations. British Journal of Psychology, 86(1), 113–125. https://doi.org/10.1111/j.2044-8295.1995.tb02549.x | D4 |
| 2 | **Kuhn** | 2008 | Kuhn, G., Tatler, B. W., Findlay, J. M., & Cole, G. G. (2008). Misdirection in magic: Implications for the relationship between eye gaze and attention. Visual Cognition, 16(2–3), 391–405. https://doi.org/10.1080/13506280701479750 | D1 |
| 3 | **Kuhn** | 2009 | Kuhn, G., Tatler, B. W., & Cole, G. G. (2009). You look where I look! Effect of gaze cues on overt and covert attention in misdirection. Visual Cognition, 17(6–7), 925–944. https://doi.org/10.1080/13506280902826775 | D1 |
| 4 | **Parris** | 2009 | Parris, B.A.; Kuhn, G.; Mizon, G.A.; Benattayallah, A.; Hodgson, T.L. (2009). Imaging the impossible: An fMRI study of impossible causal relationships in magic tricks. NeuroImage, 45(3), 1033–1039. https://doi.org/10.1016/j.neuroimage.2008.12.036 | D5 |
| 5 | **Kuhn** | 2010 | Kuhn, G.; Kourkoulou, A.; Leekam, S.R. (2010). How Magic Changes Our Expectations About Autism. Psychological Science, 21(10), 1487–1493. https://doi.org/10.1177/0956797610383435 | D1 |
| 6 | **Kuhn** | 2010 | Kuhn, G., & Findlay, J. M. (2010). Misdirection, attention and awareness: Inattentional blindness reveals temporal relationship between eye movements and visual awareness. Quarterly Journal of Experimental Psychology, 63(1), 136–146. https://doi.org/10.1080/17470210902846757 | D1 |
| 7 | **Cavina-Pratesi** | 2011 | Cavina-Pratesi, C., Kuhn, G., Ietswaart, M., & Milner, A. D. (2011). The magic grasp: Motor expertise in deception. PLOS ONE, 6(2), Article e16568. https://doi.org/10.1371/journal.pone.0016568 | D3 |
| 8 | **Cui** | 2011 | Cui, J., Otero-Millan, J., Macknik, S. L., King, M., & Martinez-Conde, S. (2011). Social misdirection fails to enhance a magic illusion. Frontiers in Human Neuroscience, 5, Article 103. https://doi.org/10.3389/fnhum.2011.00103 | D1 |
| 9 | **Rieiro** | 2013 | Rieiro, H.; Martinez-Conde, S.; Macknik, S.L. (2013). Perceptual elements in Penn & Teller's "Cups and Balls" magic trick. PeerJ, 1, e19. https://doi.org/10.7717/peerj.19 | D1 |
| 10 | **Barnhart** | 2014 | Barnhart, A. S., & Goldinger, S. D. (2014). Blinded by magic: Eye-movements reveal the misdirection of attention. Frontiers in Psychology, 5, Article 1461. https://doi.org/10.3389/fpsyg.2014.01461 | D1 |
| 11 | **Danek** | 2014 | Danek, A. H., Fraps, T., von Müller, A., Grothe, B., & Öllinger, M. (2014). Working wonders? Investigating insight with magic tricks. Cognition, 130(2), 174–185. https://doi.org/10.1016/j.cognition.2013.11.003 | D3 |
| 12 | **Tachibana** | 2014 | Tachibana, R., & Kawabata, H. (2014). The effects of social misdirection on magic tricks: How deceived and undeceived groups differ. *i-Perception, 5*(2), 143–146. https://doi.org/10.1068/i0640sas | D1 |
| 13 | **Williams** | 2014 | Williams, H., & McOwan, P. W. (2014). Magic in the machine: A computational magician's assistant. Frontiers in Psychology, 5, Article 1283. https://doi.org/10.3389/fpsyg.2014.01283 | D2 |

| No. | First author | Year | Title | Synthesis category |
| --- | --- | --- | --- | --- |
| 14 | **Danek** | 2015 | Danek, A. H., Öllinger, M., Fraps, T., Grothe, B., & Flanagin, V. L. (2015). An fMRI investigation of expectation violation in magic tricks. Frontiers in Psychology, 6, Article 84. https://doi.org/10.3389/fpsyg.2015.00084 | D5 |
| 15 | **Olson** | 2015 | Olson, J. A., Demacheva, I., & Raz, A. (2015b). Explanations of a magic trick across the life span. Frontiers in Psychology, 6, Article 219. https://doi.org/10.3389/fpsyg.2015.00219 | D3 |
| 16 | **Olson** | 2015 | Olson, J. A., Amlani, A. A., Raz, A., & Rensink, R. A. (2015a). Influencing choice without awareness. Consciousness and Cognition, 37, 225–236. https://doi.org/10.1016/j.concog.2015.01.004 | D3 |
| 17 | **Phillips** | 2015 | Phillips, F., Natter, M. B., & Egan, E. J. L. (2015). Magically deceptive biological motion—the French Drop sleight. Frontiers in Psychology, 6, 371. https://doi.org/10.3389/fpsyg.2015.00371 | D1 |
| 18 | **Caffaratti** | 2016 | Caffaratti, H., Navajas, J., Rey, H. G., & Quian Quiroga, R. (2016). Where is the ball? Behavioral and neural responses elicited by a magic trick. Psychophysiology, 53(9), 1441–1448. https://doi.org/10.1111/psyp.12691 | D5 |
| 19 | **Ekroll** | 2016 | Ekroll, V., Sayim, B., Van der Hallen, R., & Wagemans, J. (2016). Illusory visual completion of an object's invisible backside can make your finger feel shorter. Current Biology, 26(8), 1029–1033. https://doi.org/10.1016/j.cub.2016.02.001 | D2 |
| 20 | **Hergovich** | 2016 | Hergovich, A., & Oberfichtner, B. (2016). Magic and misdirection: The influence of social cues on the allocation of visual attention while watching a cups-and-balls routine. *Frontiers in Psychology, 7*, 761. https://doi.org/10.3389/fpsyg.2016.00761 | D1 |
| 21 | **Kuhn** | 2016 | Kuhn, G., Teszka, R., Tenaw, N., & Kingstone, A. (2016). Don't be fooled! Attentional responses to social cues in a face-to-face and video magic trick reveals greater top-down control for overt than covert attention. Cognition, 146, 136–142. https://doi.org/10.1016/j.cognition.2015.08.005 | D1 |
| 22 | **Kuhn** | 2016 | Kuhn, G., & Rensink, R. A. (2016). The vanishing ball illusion: A new perspective on the perception of dynamic events. Cognition, 148, 64–70. https://doi.org/10.1016/j.cognition.2015.12.003 | D2 |
| 23 | **Tamura** | 2016 | Tamura, Y., Akashi, T., Yano, S., & Osumi, H. (2016). Human visual attention model based on analysis of magic for smooth human–robot interaction. International Journal of Social Robotics, 8(5), 685–694. https://doi.org/10.1007/s12369-016-0354-y | D1 |
| 24 | **Thomas** | 2016 | Thomas, C., & Didierjean, A. (2016). Magicians fix your mind: How unlikely solutions block obvious ones. Cognition, 154, 169–173. https://doi.org/10.1016/j.cognition.2016.06.002 | D3 |
| 25 | **Tompkins** | 2016 | Tompkins, M.L.; Woods, A.T.; Aimola Davies, A.M. (2016). The Phantom Vanish Magic Trick: Investigating the Disappearance of a Non-existent Object in a Dynamic Scene. Frontiers in Psychology, 7, Article 950. https://doi.org/10.3389/fpsyg.2016.00950 | D2 |
| 26 | **Wiseman** | 2016 | Wiseman, R.J.; Nakano, T. (2016). Blink and you'll miss it: the role of blinking in the perception of magic tricks. PeerJ, 4, e1873. https://doi.org/10.7717/peerj.1873 | D1 |
| 27 | **Kawakami** | 2017 | Kawakami, N., & Miura, E. (2017). Can magic deception be detected at an unconscious level? *Perception, 46*(6), 698–708. https://doi.org/10.1177/0301006616682513 | D1 |
| 28 | **Moss** | 2017 | Moss, S.A.; Irons, M.; Boland, M. (2017). The magic of magic: The effect of magic tricks on subsequent engagement with lecture material. British Journal of Educational Psychology. https://doi.org/10.1111/bjep.12133 | D4 |
| 29 | **Rinsma** | 2017 | Rinsma, T., van der Kamp, J., Dicks, M. *et al.* Nothing magical: pantomimed grasping is controlled by the ventral system. *Exp Brain Res* **235**, 1823–1833 (2017). https://doi.org/10.1007/s00221-016-4868-1 | D3 |
| 30 | **Cocchini** | 2018 | Cocchini, G., Galligan, T., Mora, L., & Kuhn, G. (2018). The magic hand: Plasticity of mental hand representation. Quarterly Journal of Experimental Psychology, 71(11), 2314–2324. https://doi.org/10.1177/1747021817741606 | D3 |
| 31 | **Ekroll** | 2018 | Ekroll, V., De Bruyckere, E., Vanwezemael, L., & Wagemans, J. (2018). Never repeat the same trick twice—Unless it is cognitively impenetrable. i-Perception, 9(6), 1–14. https://doi.org/10.1177/2041669518816711 | D2 |
| 32 | **Ortega** | 2018 | Ortega, J., Montañes, P., Barnhart, A., & Kuhn, G. (2018). Exploiting failures in metacognition through magic: Visual awareness as a source of visual metacognition bias. Consciousness and Cognition, 65, 152–168. https://doi.org/10.1016/j.concog.2018.08.008 | D3 |
| 33 | **Thomas** | 2018 | Thomas, C.; Didierjean, A.; Kuhn, G. (2018). The Flushtration Count Illusion: Attribute substitution tricks our interpretation of a simple visual event sequence. British Journal of Psychology. https://doi.org/10.1111/bjop.12306 | D3 |
| 34 | **Barnhart** | 2019 | Barnhart, A. S., Costela, F. M., Martinez-Conde, S., Macknik, S. L., & Goldinger, S. D. (2019). Microsaccades reflect the dynamics of misdirected attention in magic. Journal of Eye Movement Research, 12(6), Article 7. https://doi.org/10.16910/jemr.12.6.7 | D1 |

| No. | First author | Year | Title | Synthesis category |
|---|---|---|---|---|
| 35 | **Gygax** | 2019 | Gygax, P., Thomas, C., Didierjean, A., & Kuhn, G. (2019). Are women perceived as worse magicians than men? Gender bias when evaluating magic tricks. Social Psychological Bulletin, 14(3), Article e33574. https://doi.org/10.32872/spb.v14i3.33574 | D3 |
| 36 | **Yao** | 2019 | Yao, R., Wood, K., & Simons, D. J. (2019). As if by magic: An abrupt change in motion direction induces change blindness. Psychological Science, 30(3), 436–443. https://doi.org/10.1177/0956797618822969 | D2 |
| 37 | **Pétervári** | 2020 | Petervari, J.; Danek, A.H. (2020). Problem solving of magic tricks: guiding to and through an impasse with solution cues. Thinking & Reasoning. https://doi.org/10.1080/13546783.2019.1668479 | D3 |
| 38 | **Quarona** | 2020 | Quarona, D., Koul, A., Ansuini, C., Pascolini, L., Cavallo, A., & Becchio, C. (2020). A kind of magic: Enhanced detection of pantomimed grasps in professional magicians. Quarterly Journal of Experimental Psychology, 73(7), 1092–1100. https://doi.org/10.1177/1747021820918533 | D3 |
| 39 | **Svalebjørg** | 2020 | Svalebjørg, M., Øhrn, H., & Ekroll, V. (2020). The illusion of absence in magic tricks. i-Perception, 11(3), 1–28. https://doi.org/10.1177/2041669520928383 | D2 |
| 40 | **Wiseman** | 2020 | Wiseman, R., Houstoun, W., & Watt, C. (2020). Pedagogic prestidigitation: Using magic tricks to enhance educational videos. PeerJ, 8, Article e9610. https://doi.org/10.7717/peerj.9610 | D4 |
| 41 | **Gronchi** | 2021 | Gronchi, G., & Zemla, J. C. (2021). Cognitive style predicts how people explain mental magic tricks. Acta Psychologica, 218, Article 103347. https://doi.org/10.1016/j.actpsy.2021.103347 | D3 |
| 42 | **Lewry** | 2021 | Lewry, C., Curtis, K., Vasilyeva, N., Xu, F., & Griffiths, T. L. (2021). Intuitions about magic track the development of intuitive physics. Cognition, 214, Article 104762. https://doi.org/10.1016/j.cognition.2021.104762 | D4 |
| 43 | **Ozono** | 2021 | Ozono, H., Komiya, A., Kuratomi, K., Hatano, A., Fastrich, G. M., Raw, J. A. L., Haffey, A., Meliss, S., Lau, J. K. L., & Murayama, K. (2021). Magic curiosity arousing tricks (MagicCATs): A novel stimulus collection to induce epistemic emotions. Behavior Research Methods, 53(1), 188–215. https://doi.org/10.3758/s13428-020-01431-2 | D4 |
| 44 | **Bagienski** | 2022 | Bagienski, S. E., Kuhn, G., & Oliver, B. R. (2022). Supporting the psychological health of our students: An arts-based community magic workshop for adapting to university life. Psychology of Consciousness: Theory, Research, and Practice, 9(3), 285–303. https://doi.org/10.1037/cns0000315 | D4 |
| 45 | **Garcia-Pelegrin** | 2022 | Garcia-Pelegrin, E., Wilkins, C., & Clayton, N. S. (2022). Investigating expert performance when observing magic effects. Scientific Reports, 12, Article 5141. https://doi.org/10.1038/s41598-022-09161-5 | D3 |
| 46 | **Lee** | 2022 | Lee, K.-T., Wang, W.-L., Lin, W.-C., Yang, Y.-C., & Tsai, C.-L. (2022). The effects of a magic intervention program on cognitive function and neurocognitive performance in elderly individuals with mild cognitive impairment. Frontiers in Aging Neuroscience, 14, Article 854984. https://doi.org/10.3389/fnagi.2022.854984 | D5 |
| 47 | **Bagienski** | 2023 | Bagienski, S. E., & Kuhn, G. (2023). A balanced view of impossible aesthetics: An empirical investigation of how impossibility relates to our enjoyment of magic tricks. i-Perception, 14(1), 1–12. https://doi.org/10.1177/20416695221142537 | D4 |
| 48 | **Begey** | 2023 | Begey, M., Didierjean, A., & Thomas, C. (2023). The Vanishing Coin Illusion: When sound congruence affects visual representation of motion. Attention, Perception, & Psychophysics, 85(6), 1768–1776. https://doi.org/10.3758/s13414-023-02776-4 | D2 |
| 49 | **Cole** | 2023 | Cole, G. G., & Millett, A. C. (2023). Visual cognition and the science of magic. Vision, 7(3), Article 56. https://doi.org/10.3390/vision7030056 | D1 |
| 50 | **Ekroll** | 2024 | Ekroll, V., Wünsch, L., & van Lier, R. (2024). Magic for the mind's eye: A promising avenue for more universal design in the art of magic. i-Perception, 15(1), 1–16. https://doi.org/10.1177/20416695231222995 | D3 |
| 51 | **Meliss** | 2024 | Meliss, S., Pascua-Martin, C., Skipper, J. I., & Murayama, K. (2024). The magic, memory, and curiosity fMRI dataset of people viewing magic tricks. Scientific Data, 11, Article 1063. https://doi.org/10.1038/s41597-024-03675-5 | D4 |
| 52 | **Plikat** | 2025 | Plikat, V., Grassi, P. R., Frack, J., & Bartels, A. (2025). Hierarchical surprise signals in naturalistic violation of expectations. Imaging Neuroscience, 3, Article imag_a_00459. https://doi.org/10.1162/imag_a_00459 | D5 |
| 53 | **Padulo** | 2026 | Padulo, C., Ponticorvo, M., & Fairfield, B. (2026). The Magic Curiosity Arousing Tricks (MagicCATs) database in Italian younger and middle-aged adults: Descriptive statistics and rule-based machine learning. Behavior Research Methods, 58, Article 1. https://doi.org/10.3758/s13428-025-02884-z | D4 |

| No. | First author | Year | Title | Synthesis category |
|---|---|---|---|---|
| 54 | **Marascia** | 2026 | Marascia, E., Di Crosta, A., La Malva, P., Ceccato, I., Prete, G., Palumbo, R., Mammarella, N., & Di Domenico, A. (2026). Exploring curiosity, interest, and surprise: Normative ratings of a magic trick video dataset in Italy. Scientific Data, 13, Article 578. https://doi.org/10.1038/s41597-026-06897-x | D4 |
| 55 | **Nguyen** | 2026 | Nguyen, A., Alexander, R. G., Venkatakrishnan, A., Macknik, S. L., & Martinez-Conde, S. (2026). Assessing the role of magician patter on deception in the Three-Card Monte. *Scientific Reports*. https://doi.org/10.1038/s41598-026-43656-9 | D1 |
